# Telomere-to-telomere, gap-free genome of mung bean (*Vigna radiata*) provides insights into domestication under structural variation

**DOI:** 10.1093/hr/uhae337

**Published:** 2024-12-02

**Authors:** Kai-Hua Jia, Guan Li, Longxin Wang, Min Liu, Zhi-Wei Wang, Ru-Zhi Li, Lei-Lei Li, Kun Xie, Yong-Yi Yang, Ru-Mei Tian, Xue Chen, Yu-Jun Si, Xiao-Yan Zhang, Feng-Jing Song, Lianzheng Li, Na-Na Li

**Affiliations:** National Saline-Alkali Tolerant Crop Germplasm Resources Nursery (Dongying), Shandong International Joint Laboratory of Agricultural Germplasm Resources Innovation, Institute of Crop Germplasm Resources, Shandong Academy of Agricultural Sciences, Jinan 250100, China; National Saline-Alkali Tolerant Crop Germplasm Resources Nursery (Dongying), Shandong International Joint Laboratory of Agricultural Germplasm Resources Innovation, Institute of Crop Germplasm Resources, Shandong Academy of Agricultural Sciences, Jinan 250100, China; School of Biological Science and Technology, University of Jinan, Jinan 250022, China; National Saline-Alkali Tolerant Crop Germplasm Resources Nursery (Dongying), Shandong International Joint Laboratory of Agricultural Germplasm Resources Innovation, Institute of Crop Germplasm Resources, Shandong Academy of Agricultural Sciences, Jinan 250100, China; National Saline-Alkali Tolerant Crop Germplasm Resources Nursery (Dongying), Shandong International Joint Laboratory of Agricultural Germplasm Resources Innovation, Institute of Crop Germplasm Resources, Shandong Academy of Agricultural Sciences, Jinan 250100, China; National Saline-Alkali Tolerant Crop Germplasm Resources Nursery (Dongying), Shandong International Joint Laboratory of Agricultural Germplasm Resources Innovation, Institute of Crop Germplasm Resources, Shandong Academy of Agricultural Sciences, Jinan 250100, China; National Saline-Alkali Tolerant Crop Germplasm Resources Nursery (Dongying), Shandong International Joint Laboratory of Agricultural Germplasm Resources Innovation, Institute of Crop Germplasm Resources, Shandong Academy of Agricultural Sciences, Jinan 250100, China; National Saline-Alkali Tolerant Crop Germplasm Resources Nursery (Dongying), Shandong International Joint Laboratory of Agricultural Germplasm Resources Innovation, Institute of Crop Germplasm Resources, Shandong Academy of Agricultural Sciences, Jinan 250100, China; National Saline-Alkali Tolerant Crop Germplasm Resources Nursery (Dongying), Shandong International Joint Laboratory of Agricultural Germplasm Resources Innovation, Institute of Crop Germplasm Resources, Shandong Academy of Agricultural Sciences, Jinan 250100, China; National Saline-Alkali Tolerant Crop Germplasm Resources Nursery (Dongying), Shandong International Joint Laboratory of Agricultural Germplasm Resources Innovation, Institute of Crop Germplasm Resources, Shandong Academy of Agricultural Sciences, Jinan 250100, China; Weifang Academy of Agricultural Sciences, Weifang 261017, China; Weifang Academy of Agricultural Sciences, Weifang 261017, China; Qingdao Academy of Agricultural Sciences, Qingdao 266100, China; Qingdao Academy of Agricultural Sciences, Qingdao 266100, China; National Saline-Alkali Tolerant Crop Germplasm Resources Nursery (Dongying), Shandong International Joint Laboratory of Agricultural Germplasm Resources Innovation, Institute of Crop Germplasm Resources, Shandong Academy of Agricultural Sciences, Jinan 250100, China; College of Biological Sciences and Technology, Beijing Forestry University, Beijing 100083, China; National Saline-Alkali Tolerant Crop Germplasm Resources Nursery (Dongying), Shandong International Joint Laboratory of Agricultural Germplasm Resources Innovation, Institute of Crop Germplasm Resources, Shandong Academy of Agricultural Sciences, Jinan 250100, China

## Abstract

Mung bean (*Vigna radiata*), an essential annual legume, holds substantial value in global agriculture due to its short growth cycle, low input requirements, and nutritional benefits. Despite extensive domestication, the genetic mechanisms underlying its morphological and physiological evolution remain incompletely understood. In this study, we present a gap-free, telomere-to-telomere genome assembly of the mung bean cultivar 'Weilv-9′, achieved through the integration of PacBio HiFi, Oxford Nanopore, and high-throughput chromosome conformation capture (Hi-C) sequencing technologies. The 500-Mb assembly, encompassing 11 chromosomes and containing 28 740 protein-coding genes, reveals that 49.17% of the genome comprises repetitive sequences. Within the genome, we found the recent amplification of transposable elements significantly impacts the expression of nearby genes. Furthermore, integrating structural variation and single-nucleotide polymorphism (SNP) data from resequencing, we identified that the fatty acid synthesis, suberin biosynthetic, and phenylpropanoid metabolic processes have undergone strong selection during domestication. These findings provide valuable insights into the genetic mechanisms driving domestication and offer a foundation for future genetic enhancement and breeding programs in mung beans and related species.

## Introduction

Mung bean (*Vigna radiata*), also known as Asia's Ancient Bean, is a significant annual, warm-season legume crop, accounting for ~8.5% of the global legume cultivation area, with >7.3 million hectares cultivated worldwide [1]. India, Myanmar, and China are the primary producers. This crop is extensively grown in Asian countries (>90%) and in the warmer regions of western countries due to its favorable attributes, such as a short growth cycle (70–90 days), low input requirements, drought tolerance, and nitrogen-fixation capabilities [2]. Mung bean seeds are nutritionally rich, containing a balanced profile of macro and micronutrients, including proteins, dietary fibers, vitamins, and minerals, as well as high levels of bioactive components [3]. Traditionally, mung beans have been consumed with cereals in China and India to complement the lysine deficiency in cereal grains. Nowadays, they are commonly used in fresh, boiled, processed, and canned food products. Their high digestible protein content makes them particularly suitable for vegetarian and vegan diets. Additionally, mung beans are an excellent protein source for children due to their low allergenic and flatulence-causing components [2]. The increasing awareness of health and the prevalence of lifestyle-related diseases globally have driven the growing demand for mung beans.

The domestication history of mung beans can be traced back to its wild progenitor, *V. radiata* var. *sublobata*, ~50 000 years ago, in the tropical and subtropical regions of the South Asian subcontinent [4]. Despite the extensive domestication process, leading to significant morphological and physiological differences between cultivated and wild types, there are virtually no reproductive barriers between them, which endows mung beans with a high degree of genetic diversity and potential for improvement [5–7]. Whole-genome resequencing, particularly targeting SNP (single-nucleotide polymorphism) variation sites, presents the most direct approach for studying domestication [8]. This method allows for a comprehensive analysis of genetic diversity and the identification of domestication-related loci. By comparing domesticated species with their wild relatives, researchers can pinpoint specific genetic changes that have occurred during domestication processes [9, 10]. Furthermore, SNP variation analysis provides insights into evolutionary dynamics, selective sweeps, and genetic bottlenecks associated with domestication events.

While SNPs have significantly advanced our understanding of domestication, structural variations (SVs), including insertions (INSs), deletions (DELs), inversions (INVs), duplications (DUP), and translocations (TRAs), offer an additional layer of genetic complexity that is equally crucial for understanding the full spectrum of genetic changes associated with domestication [11, 12]. SVs can have a more profound impact on genome structure and function than SNPs, as they often involve larger segments of DNA and can affect multiple genes or regulatory regions simultaneously. For instance, in humans, the likelihood of SVs being associated with phenotypic traits is 3-fold higher than that of SNPs [13]. They also play a vital role in domestication processes. In the context of domestication, SVs are identified as the causative genetic variants for at least one-third of known domestication alleles [14]. Numerous instances demonstrate that SV is a key driver of domestication. For example, in tomatoes, multiple SVs have been found to modify gene dosage and expression levels, thereby influencing fruit flavor, size, and yield [15]. In grapes, several SVs have been involved in coastal adaptation [16]. In soybeans, SVs have impacted the domestication of genes involved in the fatty acid biosynthesis pathway [17]. However, detailed studies on the patterns of SV variation during mung bean domestication are still limited.

Recent technological advancements in genome sequencing have facilitated the development of high-resolution reference genomes for mung bean [18–20]. However, due to the prevalence of highly tandem repetitive sequences in genomes, such as centromeric, ribosomal DNA (rDNA), and transposable element (TE) sequences, achieving complete telomere-to-telomere (T2T) genome assembly remains challenging. With advancements in sequencing technology, numerous genomic assemblies have significantly improved, reaching the T2T level. Examples include *Cucurbita maxima* [21], *Rosa rugosa* [22], *Cucumis melo* [23, 24], *Quercus variabilis* [25], and cultivated and wild soybean [17]. In this research, we produced comprehensive T2T and gap-free genome assemblies for the cultivated mung bean ‘Weilv-9’ employing an integrated approach of Pacific Biosciences (PacBio), Oxford Nanopore Technologies (ONT), and high-throughput chromosome conformation capture (Hi-C) sequencing technologies. The meticulous assemblies span 500 Mb in length, encapsulating 28 740 protein-encoding genes. By comparing with genomic data from *V. unguiculata* and *V. umbellata*, we discovered that recently amplified TEs in mung bean significantly altered the expression of nearby genes. Utilizing these T2T genome assemblies along with 34 wild and 77 cultivated high-depth resequenced mung bean datasets, comprehensive analyses integrating both SNP and SV data identified potential domestication genes linked to plant architecture transformation. Collectively, this refined mung bean genome and SVs offer unparalleled insight into the genomic shifts underpinning mung bean evolution and domestication, establishing a cornerstone for future mung bean investigations and cultivar development.

## Results

### Gap-free T2T genome assembly of mung bean

The size of the mung bean genome has previously been estimated or assembled to range from 475 to 484 Mb [18–20]. We have chosen to perform genome sequencing on ‘Weilv-9’, a mung bean variety characterized by its high yield and stress resistance, along with a compact plant form. In order to assemble the accurate and complete these genomes possible, we selected a hybrid sequencing approach. This included PacBio High-Fidelity (HiFi) long reads (46 Gb), ONT ultra-long reads (53 Gb), and Hi-C paired-end reads (64 Gb) (Supplementary Table S1). Using HiFi data, the genomic survey indicated that the genome size of Weilv-9 is ~537 Mb (Supplementary Fig. S1), which is larger than previously sequenced genomes.

**Figure 1:**
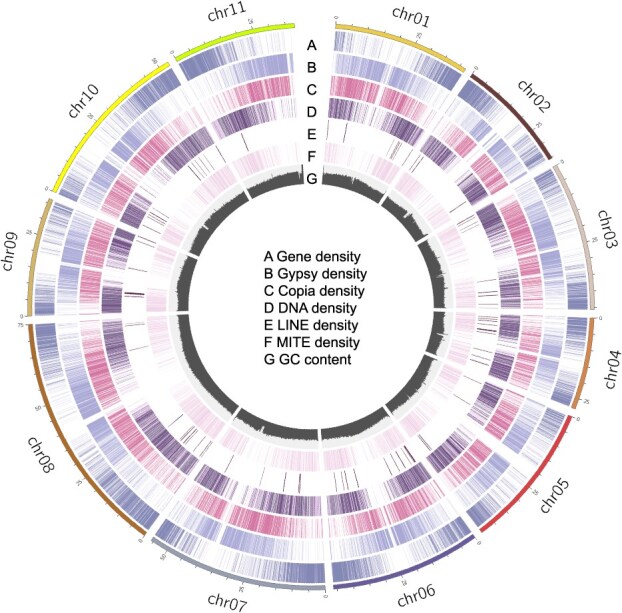
T2T genomic features of mung bean. The outermost circle displays chromosome numbers and lengths. (A) Gene density; (B) Gypsy density; (C) Copia density; (D) DNA density; (E) LINE density; (F) MITE density; (G) GC content. Counts were calculated for every 100 kb and categorized into four classes based on the count numbers, with darker colors representing higher densities.

In our initial approach, we employed the *de novo* assembler HiFiasm [26] to process HiFi, ONT, and Hi-C reads for contig generation. Subsequently, the Hi-C reads were aligned to the primary assembly utilizing Juicer [27]. This step was followed by a preliminary chromosome assembly aided by Hi-C, using 3d-DNA [28]. Manual verification and adjustments were conducted on the alignments within Juicebox [29] to ensure accuracy and correct orientation of sequences. Additionally, we undertook the reassembly of HiFi reads aligned at scaffold ends, aiming to extend their lengths. In parallel, the complete chloroplast and mitochondrial genomes were assembled using GetOrganelle [30]. The culmination of these methodologies resulted in the production of comprehensive and gap-free mung bean genome assemblies (Fig. 1). We maintained the naming and order of the assembled chromosomes consistent with previous assemblies [20]. This assembly includes 11 chromosomes along with the mitochondrial and chloroplast genomes, resulting in a total of 13 contigs, which is significantly fewer than in previous assemblies (Table 1). Collectively, these contigs span a total length of 500 144 591 bp, which is slightly smaller than the estimated genome size. The mitochondrial and chloroplast genomes measure 401 262 and 151 278 bp, respectively. The contig N50 of the mung bean genome is 46 Mb, which is also substantially greater than that of previously published mung bean genomes. Through searching for the canonical ‘TTTAGGG/CCCTAAA’ telomeric repeat, we identified 19 telomeres, with CCCTAAA at the 5′ end and TTTAGGG at the 3′ end (Supplementary Fig. S2a). The distribution of the 18–5.8-28S rDNA is primarily localized at the 5' end of chromosome 1 and the 3' end of chromosome 11 (Supplementary Fig. S2b). Conversely, the 5S rDNA shows a predominant presence on chromosomes 3, 4, and 8 (Supplementary Fig. S2c).

**Table 1:** Mung bean genome assembly statistics

Genome characteristics	VC1973A.v1	VC1973A.v2	JL7	Weilv-9
Genome size (Mb)	430.88	475.7	475.35	500.14
Contig number	25 922	1511	632	13
Scaffold number	2748	487	529	13
Number of chromosomes	11	11	11	11
N50 of contigs (Mb)	0.048	2.8	10.34	46
N50 of scaffolds (Mb)	1.52	47.1	43.79	46
BUSCO of assembled genome (%)	96.82	91.36	98.02	98.8
Number of protein-coding genes	22 427	30 958	40 125	28 740
BUSCO of protein-coding genes (%)	81.6	80.01	96.9	99.0
Annotated protein-coding genes (%)	97.9	96.2	81.6	98.5
Repeat (%)	50.13	52.79	53.45	49.17

### Comprehensive genome annotation

A total of 801 958 repetitive sequences were identified, cumulatively spanning 246 179 028 bp, which constitutes nearly half (49.17%) of the genome’s total length (Supplementary Table S2). Of these, long terminal repeat (LTR) sequences accounted for 250 426 with a combined length of 150 861 724 bp, representing 30.13% of the genome. Within the LTR category, Gypsy elements comprised 11.65%, while Copia elements constituted 8.21%. Another significant category of repetitive elements was the TIR class, occupying 8.86% of the genome.

For the comprehensive annotation of protein-coding genes in the mung bean genome, full-length transcriptome sequencing was performed. This involved pooling RNA extracted in equimolar amounts from root, stem, and leaf tissues, yielding 15G of ONT data (Supplementary Table S1). To enhance the robustness of gene annotation, 214 next-generation sequencing transcriptomic datasets were also compiled (Supplementary Table S3). After masking the repetitive regions, we employed a three-evidence-based approach for gene annotation. In total, 28 740 protein-coding genes, 1561 rRNAs, 586 tRNAs, and 843 ncRNAs (including miRNAs, tRNAs, snRNAs, and other small ncRNAs) were annotated in the mung bean genome (Table 1).

For functional annotation of these protein-coding genes, three strategies were employed. In total, 28 320 (98.54%) protein-coding genes were annotated with functional information (Table 1**,**  Supplementary Table S4). The largest proportion of functionally annotated genes was found in the TrEMBL and NR databases, accounting for 97.72% and 94.61%, respectively (Supplementary Table S4). Additionally, functional annotations were assigned to 47.65% of genes in the gene ontology (GO) database and 27.42% in the kyoto encyclopedia of genes and genomes (KEGG) pathway database.

### Assessment of T2T genome completeness

We utilized the Benchmarking Universal Single-Copy Orthologs (BUSCO) assessment with the embryophyta_odb10 lineage dataset to evaluate the completeness of the mung bean genome, revealing that out of the 1614 core genes expected, the genome showed a high level of completeness with BUSCO scores reaching 98.8% (Table 1**,**  Supplementary Table S5). In a further assessment of the protein-coding genes using BUSCO, the genome displayed an even higher completeness score, with 99.0% of the core genes being fully represented (Table 1**,**  Supplementary Table S5). Additionally, Merqury analysis, performed using a *k*-mer size of 19 based on HiFi reads, indicated a high level of genomic accuracy. The consensus quality values (QVs) were recorded at 64.26, and the *k*-mer completeness scores reached an impressive 99.73 (Table 1**,**  Supplementary Fig. S3). Moreover, when mapping Hi-C data onto the genome, no significant assembly errors were detected, indicating a high level of structural accuracy (Supplementary Fig. S4). Taken together, these various assessments underscore the exceptional quality and reliability of the mung bean genome assemblies.

### Recent LTR-RT expansion altering nearby gene expression

**Figure 2:**
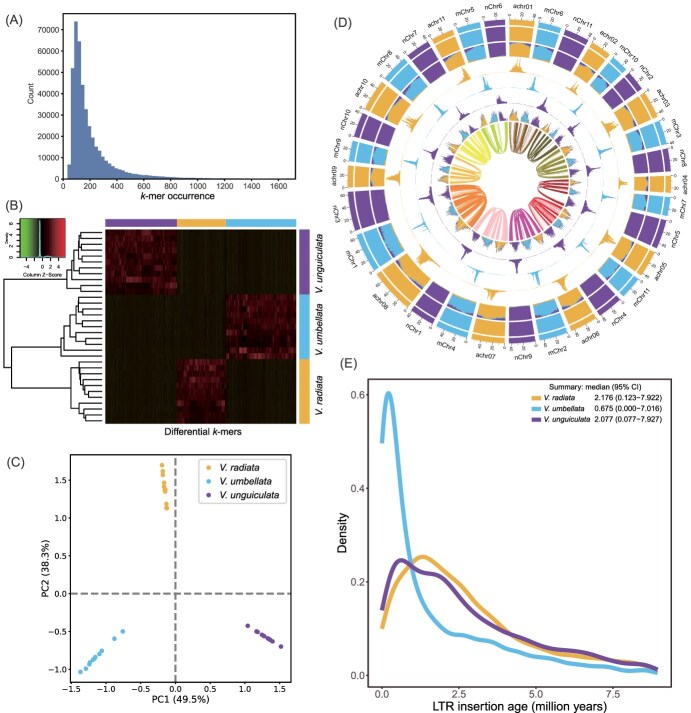
Phasing and characterization of three *Vigna* genomes. (A) The number of differential 15-mers among the homoeologous chromosomes. (B) Unsupervised hierarchical clustering is shown, with the horizontal color bar at the top indicating the specific genome of the *k*-mer, and the vertical color bar on the left indicating the assigned genome of the chromosome. The heat map displays the *Z*-scaled relative abundance of *k*-mers, with higher *Z* scores indicating greater relative abundance. (C) PCA of differential 15-mers confirms successful phasing into three genomes, based on distinct patterns of differential *k*-mers and homoeologous chromosomes. (D) Chromosomal features. From the outermost to the innermost circles (1–8) genome assignments based on a k-means algorithm; (2) significant enrichment of genome-specific k-mers, where the same color as the genome indicates significant enrichment, and white areas indicate no significant enrichment; (3) normalized proportion (relative) of genome-specific k-mers; (4–6) count (absolute) of each genome-specific k-mer set; (7) density of LTR-RTs, with consistent colors indicating significant enrichment of LTR-RTs to those genome-specific k-mers, and gray indicating nonspecific LTR-RTs; (8) homoeologous blocks. All statistics (2–7) are computed using sliding windows of 1 Mb. (E) Insertion time of genome-specific LTR-RTs, with a 95% confidence interval (CI) in the upper right corner predicting the insertion time boundaries of LTR-RTs on the genome. (b–f) Colors are consistent across genomes.

Using SubPhaser [31], we identified a total of 144 186 species-specific *k*-mer sequences based on the direct homologous relationship among the chromosomes of *V. unguiculata*, *V. umbellata*, and mung bean genomes (Fig. 2a). These species-specific *k*-mer sequences are sufficient to distinguish the three genomes (Fig. 2b–c). The majority of these species-specific *k*-mers are distributed in the central regions of the chromosomes and map to 74 772 TE sequences, with 23 980 in mung bean, 24 777 in *V. umbellata*, and 26 015 in *V. unguiculata* (Fig. 2d). Further classification of these TEs revealed that >99% of the species-specific amplified TEs are LTR retrotransposon (LTR-RT) sequences, consistent with previous findings in species such as wheat [31]. In mung bean and *V. unguiculata*, LTR expansion began ~7.9 million years ago, while in *V. umbellata*, it began ~7 million years ago, indicating their divergence ~7–8 million years ago (Fig. 2e), consistent with conclusions drawn from phylogenetic analysis [39, 40]. The evolutionary tree of LTR-RTs shows that species-specific LTR-RTs have evolved toward different directions (Supplementary Fig. S5).

**Figure 3:**
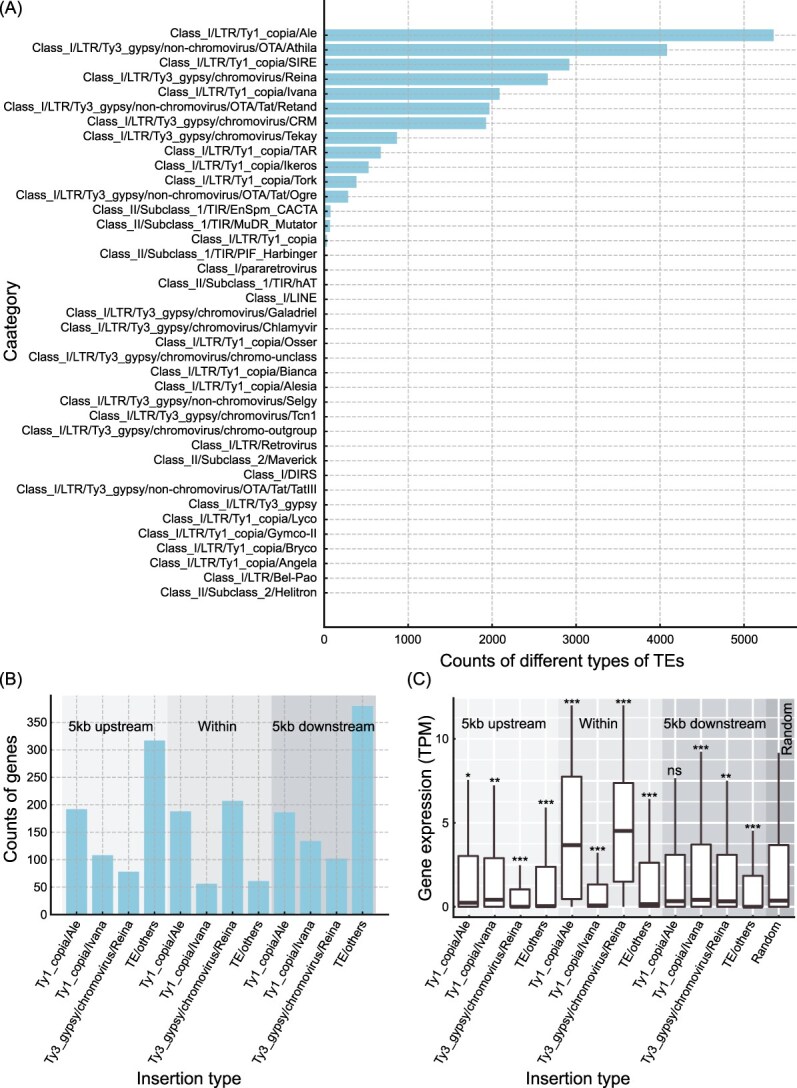
Analysis of TEs in mung bean. (A) Counts of different types of recently expanded TEs in mung bean. The x-axis represents the counts of various TEs, while the y-axis lists the different TE categories identified. (B) Distribution of different TE types within gene regions, 5 kb upstream, and 5 kb downstream. The x-axis represents the insertion type, and the y-axis represents the counts of genes associated with these insertions. (C) Gene expression levels (TPM) for genes with TE insertions within 5 kb upstream, within gene bodies, and 5 kb downstream. The box plots compare the gene expression levels with a random set of 500 genes from the genome. The significance levels indicate the statistical difference compared to the random set (^*^P < 0.05, ^**^P < 0.01, ^***^P < 0.001; Wilcoxon rank sum test).

Within the LTR class in mung bean, Ty1/copia and Ty3/gypsy were the most abundant invasions. For instance, Ale from Ty1/copia invaded 5353 times in mung bean, while Athila from Ty3/gypsy invaded 4083 times (Fig. 3a). These specific TEs inserted into 465 genes, 678 genes within the upstream 5 kb, and 791 genes within the downstream 5 kb. Interestingly, we found that the insertion of these TEs did not specifically interfere with the function of a particular category of genes, but mainly interfered with functions such as cellular hyperosmotic salinity response, benzoate metabolic process, and positive regulation of proteolysis (Supplementary Table S6–S8). Furthermore, to examine whether these different classes of TEs specifically inserted into certain positions, we categorized these TEs into four groups based on their abundance: Ty1_copia/Ale, Ty1_copia/Ivana, Ty3_gypsy/chromovirus/Reina, and TE/others. We found that regardless of whether they were inserted upstream, downstream, or within genes, Ty1_copia/Ale always had the highest number of insertions, followed by Ty1_copia/Ivana in upstream and downstream regions, but not as much within genes compared to Ty3_gypsy/chromovirus/Reina (Fig. 3b). We performed enrichment analysis on the genes affected by these four classes of TEs and found distinct patterns of insertion. The Ty3_gypsy/chromovirus/Reina primarily inserted within genes associated with the cellular response to DNA damage stimulus (Supplementary Table S9–S11). Ty1_copia/Ale mainly inserted within genes involved in vesicle-mediated transport (Supplementary Table S12–S14). Ty1_copia/Ivana predominantly inserted within genes related to inorganic ion import across the plasma membrane (Supplementary Table S15–S17). Notably, these TEs did not predominantly influence the function of specific gene categories in the upstream or downstream 5-kb regions (Supplementary Table S9–S17).

To investigate the impact of these TEs on gene expression, we downloaded a set of transcriptome data from leaf tissues of cultivated and wild mung beans (PRJNA771920) from NCBI. Our analysis revealed that TE insertions upstream, downstream, or within genes significantly altered gene expression (Fig. 3c, Wilcoxon rank sum test). For instance, Ty1_copia/Ale insertions upstream of genes significantly reduced gene expression, while insertions downstream had no effect. However, insertions within the gene body significantly increased gene expression. Ty3_gypsy/chromovirus/Reina insertions both upstream and downstream of genes significantly decreased gene expression, whereas insertions within the gene body also enhanced gene expression.

### Population structure of cultivated and wild mung bean under structural variation

We collected high-depth genomic data from 36 wild and 78 cultivated mung bean samples, with an average sequencing depth of 33.5 ± 9.7× per individual (Supplementary Table S21). These reads were mapped to the newly assembled ‘Weilv-9’ genome sequence, revealing an average coverage depth of 29×, ranging from 16.4× to 61.3×, covering 85%–98% of the genome (Supplementary Table S21). A total of 25 450 640 variant sites were identified, and after stringent filtering, 13 385 833 high-quality SNPs with a missing rate of <20% were retained. Further filtering for minor allele frequency (MAF) <0.05 resulted in 3 069 438 SNPs.

In parallel, we identified 365 831 SVs supported by at least two individuals and with a missing rate of <20%. This resulted in 115 398 retained SVs, with DELs being the most common type, comprising 90 180 variants. Additionally, there were 23 250 INSs, 1290 DUPs, and 678 INVs. These SVs spanned ~94 Mb of the genome. Inversions had the longest average length at 17057 bp, followed by duplications with an average length of 5690 bp, while insertions had the shortest average length (Supplementary Table S22).

**Figure 4:**
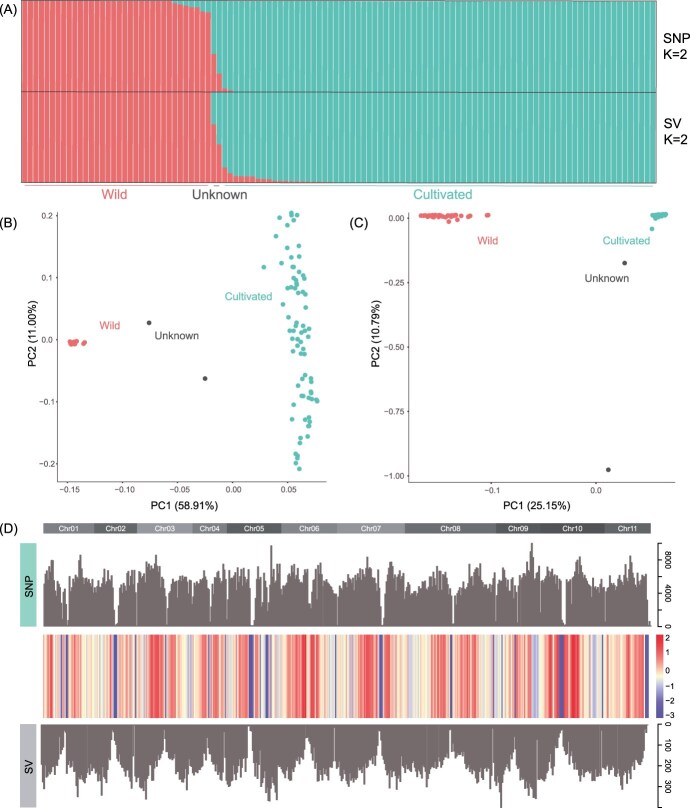
Population structure of cultivated and wild mung beans. (A) Ancestry assignment for 114 mung bean individuals based on SNPs and SVs at *K* = 2. Each bar represents an individual, with different colors indicating varying ancestry components. (B) PCA based on SNPs for cultivated and wild mung beans, with different colors representing different species/groups. (C) PCA based on SVs for cultivated and wild mung beans, with different colors representing different species/groups. (D) The residuals from a regression of SV density on SNP density (10-Mb windows), with chromosome labels above the plot. Positive residuals correspond to regions with more SVs than expected based on SNP density, while negative residuals correspond to regions with fewer SVs than expected.

Based on unlinked SNPs, our population structure analysis was consistent with previous results. At *K* = 2, the cultivated and wild populations split into two distinct groups (Fig. 4a–b), except for two individuals potentially misassigned [4]. Similarly, SV-based analysis supported these findings. Both SNP and SV analyses indicated minimal introgression between the two populations (Fig. 4a, c), unlike soybean, which shows significant introgression from cultivated to wild populations [17, 41]. After excluding the two potentially misassigned individuals, we compared the density of SNPs and SVs, finding a strong correlation (general linear model: *t*-value = 20.84, *P* < 2e-16) (Fig. 4d).

### Divergence between cultivated and wild mung bean under SVs

**Figure 5:**
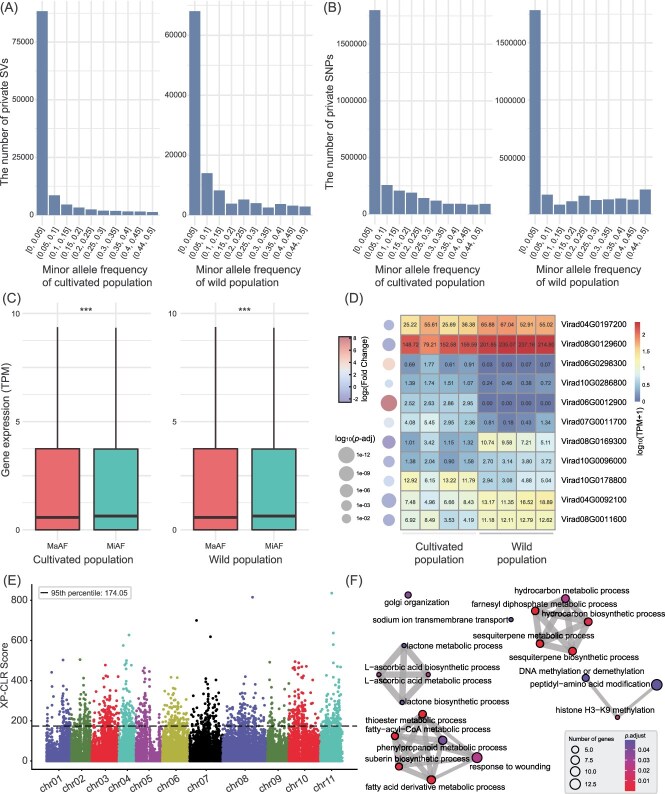
Genomic variations in cultivated and wild mung bean populations. (A) Number of private SVs in the cultivated and wild populations. (B) Number of private SNPs in the cultivated and wild populations. (C) Gene expression levels in genes affected by MiAF and MaAF SVs in both cultivated and wild populations. Asterisks indicate significant differences (*** *P* < 0.001, Wilcoxon rank sum test). (D) The expression levels of 11 significantly differentially expressed genes (*P* < 0.05, FC > 2), with TPM values indicated within the boxes. (E) Genome-wide distribution of 20-kb windows based on XP-CLR scores for SNPs. (F) Enrichment analysis of biological processes among genes affected by the intersection of the top 5% of loci with the highest XP-CLR scores and the SV dataset.

The cultivated population harbors 1 049 411 private SNPs, which impact 21 795 genes. In contrast, the wild population contains relatively fewer private SNPs (975936) compared to the cultivated group. Interestingly, we identified a higher number of private structural variants (SVs) in the wild population (51 811) than in the cultivated population (33 559). The site frequency spectrum (SFS) of these SNPs and SVs predominantly shows an MAF <0.05 (Fig. 5a–b). Generally, low-frequency SVs are likely to be deleterious or neutral and are typically not widespread in the population due to negative selection pressure, while high-frequency SVs tend to be neutral or beneficial, allowing them to persist and propagate within the population [42, 43]. Consequently, we categorized these SVs into two groups based on their MAF: 0–0.05 (Minor Allele Frequency, MiAF) and 0.05–0.5 (Major Allele Frequency, MaAF). In the cultivated population, MiAF SVs predominantly affect 7271 regions either 2 kb upstream, downstream, or within genes, whereas MaAF SVs impact 5572 genes. Similarly, in the wild population, MiAF SVs affect 8823 genes, while MaAF SVs influence 11 730 genes. We downloaded the leaf transcriptome data for cultivated mung bean and wild mung bean (SRR16477674, SRR16477675, SRR16477676, SRR16477677, SRR16477680, SRR16477686, SRR16477688, SRR16477689). Our analysis revealed that in both cultivated and wild populations, the expression levels of leaf genes impacted by MaAF SVs were significantly lower than those affected by MiAF SVs. The significance was assessed using the Wilcoxon rank sum test in R (^***^  *P* < 0.001, Fig. 5c).

To elucidate potential genes implicated in the domestication process, we aimed to identify regions of significant chromosomal divergence between wild and cultivated samples. We estimated SNP divergences within fixed 20-kb genomic windows for this purpose. The genome-wide average fixation index (*F*_ST_) estimates were substantially higher for SNPs (0.54) than for SVs (0.44), indicative of the generally lower population frequencies observed for SVs. We identified the top 5% of windows with the highest *F*_ST_ values for both SNPs and SVs and intersected these datasets, resulting in a set of 206 genes. This set includes genes involved in cell activation in immune response and defense response to symbionts (Supplementary Table S23).

Among these, 11 genes exhibited significant differential expression in leaf tissue, with five upregulated in cultivated varieties and six upregulated in wild varieties (Fig. 5d). Notably, the gene Virad08G0129600, which encodes an RNA-binding protein, has homologs in other plant species that play essential roles in stress response and chloroplast function, contributing to environmental adaptation. For example, in *Arabidopsis thaliana*, the homologous gene CP31B is crucial for chloroplast ribosome biogenesis, essential for chloroplast function and overall plant growth and development under varying conditions [44]. This suggests that Virad08G0129600 may also contribute to stress resilience in mung beans through similar mechanisms, enhancing adaptability in challenging environments. The gene Virad04G0197200 likely encodes a sodium/calcium exchanger from the Ca^2+^ cation antiporter (CaCA) family, involved in the transport of calcium and sodium ions across cellular membranes. Similar to its homologs, this protein contains EF-hand domains that facilitate calcium binding, which is essential for sensing and regulating calcium levels within the cell. Such mechanisms play a role in stress adaptation by helping maintain ionic balance under fluctuating environmental conditions, as demonstrated by homologous genes in *Oryza sativa*, where related proteins contribute to ionic homeostasis and improve plant resilience to abiotic stress [45].

### Selective pressures in the domestication of mung beans under SVs

A cross-population composite likelihood ratio (XP-CLR) test was utilized to identify species-specific selective sweeps in mung beans based on the SNP dataset. The analysis prioritized the top 5% of 20-kb windows based on the highest XP-CLR scores, resulting in 1074 regions (Fig. 5e). These regions were further intersected with SVs, yielding 382 SVs involving 350 genes. These genes were significantly enriched in fatty-acyl-CoA metabolic process, farnesyl diphosphate metabolic process, fatty acid derivative metabolic process, sesquiterpene biosynthetic and metabolic processes, and phenylpropanoid metabolic process (Fig. 5f). This suggests that fatty acid synthesis or degradation has undergone selective pressure. Additionally, processes such as suberin biosynthetic process and phenylpropanoid metabolic process were also strongly selected, indicating their critical roles in the domestication of mung beans from prostrate to erect growth forms.

## Discussion

### High-quality genome assembly and annotation as a foundation for scientific research in this species

Since the first chromosome-level genome assembly of mung bean was published in 2014 [19], three versions have been released to date [18–20]. However, these assemblies still contain numerous gaps and unanchored regions, which can hinder the identification of functional genes and impede progress in molecular breeding. Advances in sequencing and assembly technologies, particularly the advent of PacBio HiFi, ultra-long ONT, Hi-C sequencing technologies, and the hifiasm assembler [26], have enabled the creation of gap-free, T2T genomes [46]. In this study, we successfully assembled the first T2T, gap-free genome of mung bean using PacBio HiFi, ultra-long ONT, and Hi-C sequencing technologies, along with the upgraded hifiasm software. This genome assembly is also the first T2T genome for a *Vigna* species. The comprehensive assembly includes 11 chromosomes and the complete chloroplast and mitochondrial genomes, spanning a total length of 500 Mb with an impressive contig/scaffold N50 of 45 Mb. The high quality of the assembly is underscored by the BUSCO scores, indicating a completeness of 98.8% for the genome. The structural accuracy of the assembly is validated by the identification of telomeric repeats and the Hi-C interaction maps.

In addition to genome assembly, accurate gene annotation is crucial. In *Vigna stipulacea*, 30 963 protein-coding genes have been annotated [47], while in ricebean (*V. umbellata*), the number ranges from 31 276 to 37 489 [39, 48]. For Black gram (*Vigna mungo*), annotations range from 28 881 to 42 115 protein-coding genes [34, 49]. Previous mung bean genome assemblies have annotated 22 427 [19], 30 958 [18], and 40 125 [20] protein-coding genes. The coverage and continuity of the genome, annotation algorithms and tools, prediction models, and training datasets all impact the accuracy of protein-coding gene annotations [32]. To enhance annotation accuracy, we downloaded 213 next-generation RNA-seq datasets from NCBI and generated 15 Gb of full-length transcriptome data. Additionally, we incorporated 237 544 non-redundant protein sequences from species such as *V. unguiculata*, *Vigna angularis*, *V. radiata*, *Vigna subterranea*, *V. umbellata*, *Glycine max*, *Arachis hypogaea*, *Cicer arietinum*, *Medicago truncatula*, and *A. thaliana* as homologous protein evidence for gene annotation. Ultimately, the comprehensive annotation of 28 740 protein-coding genes and various non-coding RNAs enhances our understanding of the mung bean genome's functional landscape. BUSCO assessment revealed that 99.0% of our annotated protein-coding genes were complete, indicating high annotation accuracy. Additionally, using multiple databases, we achieved high-quality functional annotations for 98.54% of the genes.

The detailed gene annotation lays the groundwork for functional genomics studies aimed at elucidating the roles of specific genes and their regulatory networks. For instance, the identification of genes involved in stress responses, metabolism, and signaling pathways can guide targeted breeding efforts to develop mung bean varieties with improved stress tolerance, disease resistance, and nutritional quality. Moreover, the discovery of TEs such as Ty1_copia/Ale and Ty3_gypsy/chromovirus/Reina within gene regions and their impact on gene expression suggests that TEs play a significant role in regulating gene function and genome evolution. Understanding the mechanisms by which TEs influence gene expression and contribute to phenotypic diversity can provide new strategies for crop improvement [50, 51].

### Structural variations facilitate the domestication of cultivated mung bean

High-throughput sequencing technologies, especially those targeting SNPs, have become essential tools for studying the domestication process. Large-scale SNP analysis enables the identification of genes selected during domestication and the loci potentially lost due to artificial selection. This provides valuable insights into the genetic basis of crop domestication. However, our understanding of SVs in this context remains relatively limited. SVs, due to their complexity and size, have been significantly underestimated in their potential impact on genetic improvement [52]. SVs can cause significant changes in gene expression, affecting plant traits and adaptability. For example, during tomato domestication, certain SVs have impacted key genes regulating fruit development, leading to the evolution of diverse large-fruited varieties from small-fruited wild types [15, 53]. Similarly, in cotton improvement studies, GWAS based on SVs have identified SVs associated with fiber quality and yield [54]. Interestingly, some related genes were not identified using SNP-based GWAS, indicating that SVs and SNPs reveal different genetic information. Additionally, a tomato GWAS based on 17 flavor volatiles and 249 metabolites found only 5.2% overlap in quantitative trait loci between SVs and SNPs [55]. Another study on millet also found that SV-based GWAS improved the association efficiency for certain traits, with ~36.9% of SVs located >50 kb away from SNPs [56].

In our study, we found that the cultivated mung bean population had a significantly lower number of SVs and private SVs compared to the wild population, despite the fact that the number of wild individuals we used was half that of the cultivated individuals. This result, consistent with SNP-based findings, further supports the impact of selective pressures on genetic diversity during domestication. Furthermore, our analysis categorized these SVs into two groups based on their MAF: 0–0.05 (MiAF) and 0.05–0.5 (MaAF). In the cultivated population, MiAF SVs predominantly affect 7271 regions within 2 kb upstream or downstream of genes, whereas MaAF SVs impact 5572 genes. Similarly, in the wild population, MiAF SVs affect 8823 genes while MaAF SVs influence 11 730 genes. Notably, the expression levels of genes impacted by MaAF SVs were significantly lower than those affected by MiAF SVs in both populations, suggesting a potential regulatory role of these variants in gene expression. These findings indicate that the conservation of wild mung bean populations is critical for maintaining the genetic diversity necessary for future breeding and adaptation efforts. Wild populations serve as a genetic reservoir that can be tapped into to enhance the genetic robustness of cultivated varieties. Protecting these natural habitats and preventing genetic erosion through conservation strategies will ensure the availability of diverse genetic material for sustainable agriculture.

To elucidate potential genes implicated in the domestication process, we identified regions of significant chromosomal divergence between wild and cultivated samples. Using *F*_ST_ estimates within fixed 20-kb genomic windows, we found that the genome-wide average *F*_ST_ was substantially higher for SNPs (0.54) than for SVs (0.44). This suggests a generally lower population frequency of SVs compared to SNPs. By intersecting the top 5% of windows with the highest *F*_ST_ for SNPs with the SV dataset, we identified 206 genes involved in immune-related functions, some of which exhibited significant differential expression between the two populations. Future research should focus on characterizing the specific genomic regions affected by domestication and the functional implications of these changes. Investigating the interaction between SNPs and SVs in shaping phenotypic diversity will provide deeper insights into the genetic mechanisms underlying adaptation and domestication. Additionally, studies exploring the epigenetic modifications associated with structural variants could reveal further layers of regulation that contribute to the observed differences in gene expression.

### Role of suberin and phenylpropanoid pathways in structural and defense adaptations

The XP-CLR analysis identified 1074 regions in mung beans, with 382 SVs involving 350 genes, significantly enriched in pathways such as fatty-acyl-CoA metabolic process, farnesyl diphosphate metabolic process, and phenylpropanoid metabolic process. This suggests selective pressures on fatty acid metabolism and secondary metabolite production, essential for plant defense and structural integrity. The strong selection on suberin and phenylpropanoid pathways indicates their role in the transition from prostrate to erect growth forms, enhancing physical strength and pathogen resistance.

Suberin, a biopolymer composed of phenolic and fatty acid derivatives, is primarily deposited in the root bark, seed coats, and tuber skins of plants. It plays a vital role in preventing water loss and pathogen invasion in the plant cell wall [57]. In cultivated mung beans, the selective expression of genes related to suberin biosynthesis may have contributed to increased physical strength, aiding the transition from prostrate growth to erect growth forms. Phenylpropanoid metabolic products, including lignin and flavonoids, are crucial for plant structure and defense. Lignin provides physical support and stress resistance in the cell wall, while flavonoids offer antioxidant and antimicrobial properties [58]. In mung beans, the selective pressure on genes involved in the phenylpropanoid metabolic process may have enhanced structural stability and disease resistance, promoting the transition from prostrate to erect growth. Studies in maize, poplar, and rice have shown similar impacts of lignin biosynthesis gene mutations on plant mechanical properties and disease resistance, supporting the importance of these pathways in mung bean domestication [59–62].

## Methods

### Plant materials and sequencing

A new variety of mung bean, Weilv-9, developed by the Agricultural Institute of Weifang, Shandong Province, was germinated in the laboratory. Fresh leaves were harvested for DNA extraction using a modified CTAB method as previously described [63].

Three sequencing approaches were employed to analyze the genomic DNA. Initially, DNA integrity was assessed using Femto Pulse, followed by library preparation with the Pacific Biosciences SMRTbell Express Template Prep Kit 2.0, and sequencing was conducted on the PacBio Sequel II platform. Additionally, DNA fragments that passed quality control were utilized for library construction using the Oxford Nanopore SQK-LSK109 kit and were sequenced on the PromethION sequencer (Oxford Nanopore Technologies, Oxford, UK) using R9.4 flow cells. Finally, Hi-C libraries were generated following the protocol by Wang et al. [64] and sequenced using the DNBSEQ platform.

### *De novo* genome assembly

FindGSE [65] was utilized to conduct a genomic survey. The genome was assembled utilizing a hybrid strategy that incorporated PacBio HiFi, ONT, and Hi-C sequencing reads. Initial contigs were constructed using hifiasm v0.19.8-r602 [26]. Chromosomal-scale scaffolding commenced with the alignment of Hi-C reads to the primary assembly using Juicer v1.6 [27]. This was followed by clustering with 3D-DNA v180922 [28] and manual refinement of potential mis-assemblies using Juicebox v1.11.08 [29]. Telomere sequences were extended by realigning HiFi reads to the chromosomal ends, thus extending the scaffolds. Organelle genomes were independently assembled using GetOrganelle [30]. The final genome assembly was polished with a single round of NextPolish v1.3.1 [66] using short reads. To eliminate redundancies and contaminants, unplaced contigs were aligned to the chromosomal and organelle assemblies using redundans v0.13c [67] to identify and subsequently remove haplotigs, organellar fragments, and rDNA fragments through manual inspection. This integrated approach of long-read sequencing and Hi-C scaffolding culminated in a robust, high-quality, chromosome-scale reference genome.

### Repeat identification

TEs were *de novo* identified from the genome using the EDTA v1.9.9 [68]. The analysis was conducted under sensitive settings (--sensitive 1 --anno 1) to develop a comprehensive TE library. This library served as a reference for RepeatMasker [69], which facilitated the identification and annotation of repetitive elements within the genome. Using the consensus sequences and profile hidden Markov models from the EDTA-generated library, RepeatMasker executed sequence alignments to classify and delineate genomic repeats, including low-complexity DNA, satellite sequences, simple repeats, and transposable elements.

### Transcriptome data sequencing and collection

To elucidate the transcriptomic profile of wild mung bean, nanopore sequencing was employed on a pooled sample consisting of roots, stems, and leaves, generating 15 G of full-length cDNA reads. Additionally, this analysis was enhanced by incorporating 214 high-quality short-read RNA-seq datasets sourced from the NCBI Sequence Read Archive.

### Gene prediction and function annotation

The genome was annotated by leveraging evidence from homologous proteins, transcript data, and *ab initio* predictions. Homologous protein evidence was gathered by searching against a collection of 237 544 non-redundant protein sequences from 10 plant species, including *Vigna* and *Glycine* genera, among others. Transcript evidence was procured through alignment of both short and long RNA-seq reads to the genome, using Hisat2 v2.1.0 [70] for short reads and minimap2 v2.24-r1122 [71] for long reads, followed by assembly with StringTie v1.3.5 [72].

Gene structures were annotated using PASA v2.4.1 [73], which utilized transcript evidence. Full-length genes were identified by comparing them to reference proteins. AUGUSTUS v3.4.0 [74] was then trained and optimized for gene prediction using the identified full-length gene set. The MAKER v2.31.9 [38] annotation pipeline integrated *ab initio* predictions, transcript, and protein evidence. EvidenceModeler (EVM) v1.1.1 [75] was employed to integrate and refine the MAKER and PASA annotations, with TEsorter [76] used to mask TE protein domains. Abnormal gene models and those <50 amino acids were excluded.

Non-coding RNA (ncRNA) annotations were performed using tRNAScan-SE v1.3.1 [77], barrnap (https://github.com/tseemann/barrnap,), and RfamScan [78].

Functional annotations of protein-coding genes were executed using three complementary approaches. First, alignment to the eggNOG v5.0 ortholog database using eggNOG-mapper v2.0.1 [37] provided GO and KEGG annotations. Secondly, similarity searches against protein databases such as SwissProt, TrEMBL, and others were conducted with diamond v0.9.24 [36], identifying best-matching orthologs based on sequence identity and E-value criteria. Finally, domain architecture analysis was carried out by scanning against Hidden Markov Models in the InterPro database using InterProScan v5.27–66.0 [79]. These homology-based methods collectively facilitated a comprehensive functional annotation of the protein-coding genes, linking them to known sequences and conserved domains.

### Assessment of genome completeness and protein-coding genes

The genome assembly's quality was assessed through three distinct methodologies. Initially, the completeness of the genome sequences and protein-coding gene sets was evaluated using BUSCO v2.0.1 [80], which quantifies the presence of conserved core genes across the assembly. Subsequently, the assembly's fidelity was gauged using Merqury [81], which employs a *k*-mer size of 19 to evaluate genome accuracy. Lastly, to detect potential assembly errors, Hi-C data was mapped onto the assembled genome using Juicer, providing insights into the structural integrity of the genome.

### Genome comparison and species-specific sequence identification

SubPhaser v1.0.5 [31] was employed to identify differential repeats across the three *Vigna* genomic assemblies, including *V. umbellate* [82], *V. unguiculata* [83], and the mung bean genome sequenced in this manuscript. Bedtools [84] was used to ascertain the intersections between functional genes and specific repetitive sequences.

### Transcriptome data analysis

Fastp v0.12.4 [85] is utilized to eliminate short reads (<60 bp), potential adapter sequences, and low-quality reads from the sequencing data. Following this preprocessing, gene expression levels are quantified in transcripts per million (TPM) using salmon v1.6.0 [86], which incorporates advanced options such as --validateMappings and --numBootstraps 100 to ensure accurate mapping and estimation of expression variability. The processed data is then further analyzed with tximport v1.22.0 [87] for downstream bioinformatics evaluations. DESeq2 v1.34.0 [88] was used to perform differential gene expression analysis. Prior to the analysis, genes with TPM values <1 across all samples were excluded.

### Collection and SNP calling of whole-genome resequencing data

A total of 114 mung bean accessions, including 36 wild and 78 cultivated varieties, were downloaded from NCBI's project PRJNA838242 [4]. Paired-end reads were processed using fastp v0.23.2 to trim adapters, remove low-quality bases, and discard short reads (<60 bp). These filtered reads were then aligned to the ‘Weilv-9’ reference genome using BWA-MEM [89]. SNP calling was carried out with Freebayes [90]. To minimize bias in SNP calling and ensure only high-quality SNPs were retained, we implemented the following filtering steps: (1) SNPs with a quality score <20 were removed; (2) only bi-allelic SNPs were retained; (3) depth parameters were set to --minDP 5 and --maxDP 500; (4) SNPs with a missing rate >20% were removed; (5) all SNPs with an MAF <0.05 were also excluded.

### SV discovery

In Dysgu v1.6.3 [91], each BAM file was processed using the dysgu run command to identify SVs, retaining only those flagged as ‘PASS’. The filtered VCF files from all samples were then merged using the dysgu merge command. Subsequently, a second round of SV calling was performed on the merged VCF using dysgu run –sites, with only variants meeting the established filtering criteria retained. The final consolidated VCF file was generated by merging the filtered VCF files from all individuals once again using dysgu merge. We then removed variants with a missing rate >20%, those with a minimum allele count less than two, and SVs >1 Mb following [16].

### Examining SNP and SV density patterns

To investigate the correlations between SNP and SV densities throughout the mung bean genome, we utilized tabix v1.9 [92] to quantify variants within 1-Mb intervals across all 20 chromosomes. We conducted correlation analyses using the glm() function, specifically focusing on the residuals retained from these binned variant densities. The overlap among different genomic regions was assessed using the bedtools intersect function.

### Population structure and divergence

We utilized ADMIXTURE v1.3.0 [33] for population structure analysis and GCTA v1.94.1 [93] for principal component analysis (PCA). Visualization was conducted through clumak [94] and ggplot2 v3.4.2 [95]. We utilized vcftools v0.1.16 [96] to calculate *F*_ST_, allele frequencies, and private alleles.

### Putative selective sweeps

Comprehensive genome analysis was conducted employing XP-CLR [97], a technique predicated on the probability modeling of multilocus allele frequency divergence across two distinct populations. Following phasing with Beagle v5.4 [35], the XP-CLR program was executed for each chromosome with specified parameters: --ld 0.95 --phased.

### GO enrichment

GO enrichment was executed via R’s clusterProfiler v4.2.2 package [98]. Key steps included initial GO term identification using ‘enrichGO’. Redundant terms were minimized using the ‘simplify’ function, followed by interterm similarity assessment with the ‘pairwise_termsim’ method employing the Jaccard coefficient. Visualization of the intricate GO term network was achieved with enrichplot v1.14.2 [99].

## Supplementary Material

Web_Material_uhae337

## Data Availability

The whole genome sequence data reported in this paper have been deposited in the Genome Sequence Archive, under accession number PRJCA021300. The genome assembly and annotation data reported in this paper have been deposited in the Genome Warehouse in National Genomics Data Center, Beijing Institute of Genomics, Chinese Academy of Sciences/China National Center for Bioinformation, under accession number GWHEQVC00000000 that is publicly accessible at https://ngdc.cncb.ac.cn/gwh.

## Aknowledgement

We extend our gratitude to the Modern Agriculture Coarse Grain Industry Technology System of Shandong Province for their valuable suport. This research was supported by the Key R&D Program of Shandong Province (2022LZGC022, 2021LZGC025, and 2023LZGC001), the National Natural Science Foundation of China (32201736), the Natural Science Foundation of Shandong Province for Young Scholars (ZR2023QC153), and the Agricultural Science and Technology Innovation Project of SAAS (CXGC2023F13, CXGC2024F13, and CXGC2023C02).

## References

[1] Nair R, Schreinemachers P. Global status and economic importance of mungbean. Mungbean Genome. 2020;1:1–8

[2] Ha J, Lee S-H. Mung bean (Vigna radiata (L.) R. Wilczek) breeding. In: Al-Khayri J, Jain S, Johnson D, eds. Advances in Plant Breeding Strategies: Legumes. Springer: Cham, 2019,371–407

[3] Hou D, Yousaf L, Xue Y. et al. Mung bean (*Vigna radiata* L.): bioactive polyphenols, polysaccharides, peptides, and health benefits. Nutrients. 2019;11:1238 31159173 10.3390/nu11061238 PMC6627095

[4] Lin YP, Chen HW, Yeh PM. et al. Demographic history and distinct selection signatures of two domestication genes in mungbean. Plant Physiol. 2023;193:1197–212 37335936 10.1093/plphys/kiad356

[5] Ignacimuthu S, Babu C. *Vigna radiata* var. *sublobata* (Fabaceae): economically useful wild relative of urd and mung beans. Econ Bot. 1987;41:418–22

[6] Pandiyan M, Senthil N, Ramamoorthi N. et al. Interspecific hybridization of *Vigna radiata* x 13 wild *Vigna* species for developing MYMV donar. Electron J Plant Breed. 2010;1:600–10

[7] Thuan ND . Expression and Inheritance of Traits in Wild Mungbean (Vigna Radiata Ssp. Sublobata) x Cultivated Mungbean (V. Radiata Ssp. Radiata) Hybrids. James Cook University; 2011:

[8] Xu X, Bai G. Whole-genome resequencing: changing the paradigms of SNP detection, molecular mapping and gene discovery. Mol Breed. 2015;35:33

[9] Nan J, Ling Y, An J. et al. Genome resequencing reveals independent domestication and breeding improvement of naked oat. Gigascience. 2022;12:giad061 37524540 10.1093/gigascience/giad061 PMC10390318

[10] Wu D, Xie L, Sun Y. et al. A syntelog-based pan-genome provides insights into rice domestication and de-domestication. Genome Biol. 2023;24:179 37537691 10.1186/s13059-023-03017-5 PMC10401782

[11] Cumer T, Boyer F, Pompanon F. Genome-wide detection of structural variations reveals new regions associated with domestication in small ruminants. Genome Biol Evol. 2021;13:evab165 34264322 10.1093/gbe/evab165 PMC8350358

[12] Hitte C . Toward the identification and role of structural variations during dog domestication. Natl Sci Rev. 2019;6:123–4 10.1093/nsr/nwy086 PMC8291424 34691836

[13] Chaisson MJP, Sanders AD, Zhao X. et al. Multi-platform discovery of haplotype-resolved structural variation in human genomes. Nat Commun. 2019;10:1784 30992455 10.1038/s41467-018-08148-z PMC6467913

[14] Gaut BS, Seymour DK, Liu Q. et al. Demography and its effects on genomic variation in crop domestication. Nat Plants. 2018;4:512–20 30061748 10.1038/s41477-018-0210-1

[15] Alonge M, Wang X, Benoit M. et al. Major impacts of widespread structural variation on gene expression and crop improvement in tomato. Cell. 2020;182:145–161.e23 32553272 10.1016/j.cell.2020.05.021 PMC7354227

[16] Zhang T, Peng W, Xiao H. et al. Population genomics highlights structural variations in local adaptation to saline coastal environments in woolly grape. J Integr Plant Biol. 2024;66:1408–26 38578160 10.1111/jipb.13653

[17] Jia KH, Zhang X, Li LL. et al. Telomere-to-telomere genome assemblies of cultivated and wild soybean provide insights into evolution and domestication under structural variation. Plant Commun. 2024;5:100919 38605518 10.1016/j.xplc.2024.100919 PMC11369727

[18] Ha J, Satyawan D, Jeong H. et al. A near-complete genome sequence of mungbean (*Vigna radiata* L.) provides key insights into the modern breeding program. Plant Genome. 2021;14:e20121 34275211 10.1002/tpg2.20121 PMC12807378

[19] Kang YJ, Kim SK, Kim MY. et al. Genome sequence of mungbean and insights into evolution within *Vigna* species. Nat Commun. 2014;5:5443 25384727 10.1038/ncomms6443 PMC4241982

[20] Liu C, Wang Y, Peng J. et al. High-quality genome assembly and pan-genome studies facilitate genetic discovery in mung bean and its improvement. Plant Commun. 2022;3:100352 35752938 10.1016/j.xplc.2022.100352 PMC9700124

[21] Zeng Q, Wei M, Li S. et al. Complete genome assembly provides insights into the centromere architecture of pumpkin (*Cucurbita maxima*). Plant Commun. 2024;5:100935 38689498 10.1016/j.xplc.2024.100935 PMC11412922

[22] Liu D, Liu K, Tong B. et al. Telomere-to-telomere, gap-free assembly of the *Rosa rugosa* reference genome. Hortic Plant J. 2024

[23] Mo C, Wang H, Wei M. et al. Complete genome assembly provides a high-quality skeleton for pan-NLRome construction in melon. Plant J. 2024;118:2249–68 38430487 10.1111/tpj.16705

[24] Wei M, Huang Y, Mo C. et al. Telomere-to-telomere genome assembly of melon (*Cucumis melo* L. var. inodorus) provides a high-quality reference for meta-QTL analysis of important traits. Hortic Res. 2023;10:uhad189 37915500 10.1093/hr/uhad189 PMC10615816

[25] Wang L, Li LL, Chen L. et al. Telomere-to-telomere and haplotype-resolved genome assembly of the Chinese cork oak (*Quercus variabilis*). Front Plant Sci. 2023;14:1290913 38023918 10.3389/fpls.2023.1290913 PMC10652414

[26] Cheng H, Concepcion GT, Feng X. et al. Haplotype-resolved *de novo* assembly using phased assembly graphs with hifiasm. Nat Methods. 2021;18:170–5 33526886 10.1038/s41592-020-01056-5 PMC7961889

[27] Durand NC, Shamim MS, Machol I. et al. Juicer provides a one-click system for analyzing loop-resolution Hi-C experiments. Cell Syst. 2016;3:95–8 27467249 10.1016/j.cels.2016.07.002 PMC5846465

[28] Dudchenko O, Batra SS, Omer AD. et al. *De novo* assembly of the *Aedes aegypti* genome using Hi-C yields chromosome-length scaffolds. Science. 2017;356:92–5 28336562 10.1126/science.aal3327 PMC5635820

[29] Robinson JT, Turner D, Durand NC. et al. Juicebox.js provides a cloud-based visualization system for Hi-C data. Cell Syst. 2018;6:256–258.e1 29428417 10.1016/j.cels.2018.01.001 PMC6047755

[30] Jin JJ, Yu WB, Yang JB. et al. GetOrganelle: a fast and versatile toolkit for accurate *de novo* assembly of organelle genomes. Genome Biol. 2020;21:241 32912315 10.1186/s13059-020-02154-5 PMC7488116

[31] Jia KH, Wang ZX, Wang L. et al. SubPhaser: a robust allopolyploid subgenome phasing method based on subgenome-specific *k*-mers. New Phytol. 2022;235:801–9 35460274 10.1111/nph.18173

[32] Abbas Q, Wilhelm M, Kuster B. et al. Exploring crop genomes: assembly features, gene prediction accuracy, and implications for proteomics studies. BMC Genomics. 2024;25:619 38898442 10.1186/s12864-024-10521-w PMC11186247

[33] Alexander DH, Novembre J, Lange K. Fast model-based estimation of ancestry in unrelated individuals. Genome Res. 2009;19:1655–64 19648217 10.1101/gr.094052.109 PMC2752134

[34] Ambreen H, Oraon PK, Wahlang DR. et al. Long-read-based draft genome sequence of Indian black gram IPU-94-1 'Uttara': insights into disease resistance and seed storage protein genes. Plant Genome. 2022;15:e20234 35762493 10.1002/tpg2.20234 PMC12807400

[35] Browning SR, Browning BL. Rapid and accurate haplotype phasing and missing-data inference for whole-genome association studies by use of localized haplotype clustering. Am J Hum Genet. 2007;81:1084–97 17924348 10.1086/521987 PMC2265661

[36] Buchfink B, Xie C, Huson DH. Fast and sensitive protein alignment using DIAMOND. Nat Methods. 2015;12:59–60 25402007 10.1038/nmeth.3176

[37] Cantalapiedra CP, Hernández-Plaza A, Letunic I. et al. eggNOG-mapper v2: functional annotation, orthology assignments, and domain prediction at the metagenomic scale. Mol Biol Evol. 2021;38:5825–9 34597405 10.1093/molbev/msab293 PMC8662613

[38] Cantarel BL, Korf I, Robb SM. et al. MAKER: an easy-to-use annotation pipeline designed for emerging model organism genomes. Genome Res. 2008;18:188–96 18025269 10.1101/gr.6743907 PMC2134774

[39] Francis A, Singh NP, Singh M. et al. The ricebean genome provides insight into *Vigna* genome evolution and facilitates genetic enhancement. Plant Biotechnol J. 2023;21:1522–4 37323100 10.1111/pbi.14075 PMC10363756

[40] Liang L, Zhang J, Xiao J. et al. Genome and pan-genome assembly of asparagus bean (*Vigna unguiculata* ssp. *sesquipedialis*) reveal the genetic basis of cold adaptation. Front Plant Sci. 2022;13:1059804 36589110 10.3389/fpls.2022.1059804 PMC9802904

[41] Wang X, Chen L, Ma J. Genomic introgression through interspecific hybridization counteracts genetic bottleneck during soybean domestication. Genome Biol. 2019;20:1–15 30700312 10.1186/s13059-019-1631-5 PMC6354408

[42] Pollex RL, Hegele RA. Copy number variation in the human genome and its implications for cardiovascular disease. Circulation. 2007;115:3130–8 17576883 10.1161/CIRCULATIONAHA.106.677591

[43] Sudmant PH, Rausch T, Gardner EJ. et al. An integrated map of structural variation in 2,504 human genomes. Nature. 2015;526:75–81 26432246 10.1038/nature15394 PMC4617611

[44] Kupsch C, Ruwe H, Gusewski S. et al. *Arabidopsis* chloroplast RNA binding proteins CP31A and CP29A associate with large transcript pools and confer cold stress tolerance by influencing multiple chloroplast RNA processing steps. Plant Cell. 2012;24:4266–80 23110894 10.1105/tpc.112.103002 PMC3517249

[45] Teng X-X, Cao W-L, Lan H-X. et al. OsNHX2, an Na+/H+ antiporter gene, can enhance salt tolerance in rice plants through more effective accumulation of toxic Na+ in leaf mesophyll and bundle sheath cells. Acta Physiol Plant. 2017;39:1–8

[46] Li H, Durbin R. Genome assembly in the telomere-to-telomere era. Nat Rev Genet. 2024;1:1–13 10.1038/s41576-024-00718-w 38649458

[47] Takahashi Y, Sakai H, Ariga H. et al. Domesticating *Vigna* stipulacea: chromosome-level genome assembly reveals VsPSAT1 as a candidate gene decreasing hard-seededness. Front Plant Sci. 2023;14:1119625 37139108 10.3389/fpls.2023.1119625 PMC10149957

[48] Kaul T, Easwaran M, Thangaraj A. et al. *De novo* genome assembly of rice bean (*Vigna umbellata*) - a nominated nutritionally rich future crop reveals novel insights into flowering potential, habit, and palatability centric - traits for efficient domestication. Front Plant Sci. 2022;13:739654 36267942 10.3389/fpls.2022.739654 PMC9577371

[49] Jegadeesan S, Raizada A, Dhanasekar P. et al. Draft genome sequence of the pulse crop blackgram [*Vigna mungo* (L.) Hepper] reveals potential R-genes. Sci Rep. 2021;11:11247 34045617 10.1038/s41598-021-90683-9 PMC8160138

[50] Hassan AH, Mokhtar MM, El Allali A. Transposable elements: multifunctional players in the plant genome. Front Plant Sci. 2023;14:1330127 38239225 10.3389/fpls.2023.1330127 PMC10794571

[51] He X, Qi Z, Liu Z. et al. Pangenome analysis reveals transposon-driven genome evolution in cotton. BMC Biol. 2024;22:92 38654264 10.1186/s12915-024-01893-2 PMC11040754

[52] Scott AJ, Chiang C, Hall IM. Structural variants are a major source of gene expression differences in humans and often affect multiple nearby genes. Genome Res. 2021;31:2249–57 34544830 10.1101/gr.275488.121 PMC8647827

[53] Jobson E, Roberts R. Genomic structural variation in tomato and its role in plant immunity. Mol Hortic. 2022;2:7 37789472 10.1186/s43897-022-00029-w PMC10515242

[54] He S, Sun G, Geng X. et al. The genomic basis of geographic differentiation and fiber improvement in cultivated cotton. Nat Genet. 2021;53:916–24 33859417 10.1038/s41588-021-00844-9

[55] Li N, He Q, Wang J. et al. Super-pangenome analyses highlight genomic diversity and structural variation across wild and cultivated tomato species. Nat Genet. 2023;55:852–60 37024581 10.1038/s41588-023-01340-y PMC10181942

[56] He Q, Tang S, Zhi H. et al. A graph-based genome and pan-genome variation of the model plant *Setaria*. Nat Genet. 2023;55:1232–42 37291196 10.1038/s41588-023-01423-w PMC10335933

[57] Nomberg G, Marinov O, Arya GC. et al. The key enzymes in the suberin biosynthetic pathway in plants: an update. Plan Theory. 2022;11:392 10.3390/plants11030392 PMC8839845 35161373

[58] Xin A, Herburger K. Precursor biosynthesis regulation of lignin, suberin and cutin. Protoplasma. 2021;258:1171–8 34120228 10.1007/s00709-021-01676-4

[59] Ma QH . Lignin biosynthesis and its diversified roles in disease resistance. Genes (Basel). 2024;15:295 38540353 10.3390/genes15030295 PMC10969841

[60] Qin S, Fan C, Li X. et al. LACCASE14 is required for the deposition of guaiacyl lignin and affects cell wall digestibility in poplar. Biotechnol Biofuels Bioprod. 2020;13:197 10.1186/s13068-020-01843-4 PMC7713150 33292432

[61] Wang Y, Wang M, Yan X. et al. The DEP1 mutation improves stem lodging resistance and biomass saccharification by affecting cell wall biosynthesis in rice. Rice (N Y). 2024;17:35 38748282 10.1186/s12284-024-00712-0 PMC11096150

[62] Xiong W, Wu Z, Liu Y. et al. Mutation of 4-coumarate: coenzyme a ligase 1 gene affects lignin biosynthesis and increases the cell wall digestibility in maize brown midrib5 mutants. Biotechnol Biofuels Bioprod. 2019;12:82 10.1186/s13068-019-1421-z PMC6456989 31007716

[63] Doyle JJ, Doyle JL. A rapid DNA isolation procedure for small quantities of fresh leaf tissue. Phytochem Bull. 1987;19:11–5

[64] Wang C, Liu C, Roqueiro D. et al. Genome-wide analysis of local chromatin packing in *Arabidopsis thaliana*. Genome Res. 2015;25:246–56 25367294 10.1101/gr.170332.113 PMC4315298

[65] Sun H, Ding J, Piednoël M. et al. findGSE: estimating genome size variation within human and *Arabidopsis* using *k*-mer frequencies. Bioinformatics. 2018;34:550–7 29444236 10.1093/bioinformatics/btx637

[66] Hu J, Fan J, Sun Z. et al. NextPolish: a fast and efficient genome polishing tool for long-read assembly. Bioinformatics. 2020;36:2253–5 31778144 10.1093/bioinformatics/btz891

[67] Pryszcz LP, Gabaldon T. Redundans: an assembly pipeline for highly heterozygous genomes. Nucleic Acids Res. 2016;44:e113 27131372 10.1093/nar/gkw294 PMC4937319

[68] Ou S, Su W, Liao Y. et al. Benchmarking transposable element annotation methods for creation of a streamlined, comprehensive pipeline. Genome Biol. 2019;20:275 31843001 10.1186/s13059-019-1905-y PMC6913007

[69] Chen N . Using RepeatMasker to identify repetitive elements in genomic sequences. Curr Protoc Bioinformatics. 2004;Chapter 4:4.10.1–14 10.1002/0471250953.bi0410s05 18428725

[70] Kim D, Langmead B, Salzberg SL. HISAT: a fast spliced aligner with low memory requirements. Nat Methods. 2015;12:357–60 25751142 10.1038/nmeth.3317 PMC4655817

[71] Li H . Minimap2: pairwise alignment for nucleotide sequences. Bioinformatics. 2018;34:3094–100 29750242 10.1093/bioinformatics/bty191 PMC6137996

[72] Pertea M, Pertea GM, Antonescu CM. et al. StringTie enables improved reconstruction of a transcriptome from RNA-seq reads. Nat Biotechnol. 2015;33:290–5 25690850 10.1038/nbt.3122 PMC4643835

[73] Haas BJ, Delcher AL, Mount SM. et al. Improving the *Arabidopsis* genome annotation using maximal transcript alignment assemblies. Nucleic Acids Res. 2003;31:5654–66 14500829 10.1093/nar/gkg770 PMC206470

[74] Stanke M, Diekhans M, Baertsch R. et al. Using native and syntenically mapped cDNA alignments to improve *de novo* gene finding. Bioinformatics. 2008;24:637–44 18218656 10.1093/bioinformatics/btn013

[75] Haas BJ, Salzberg SL, Zhu W. et al. Automated eukaryotic gene structure annotation using EVidenceModeler and the program to assemble spliced alignments. Genome Biol. 2008;9:R7 18190707 10.1186/gb-2008-9-1-r7 PMC2395244

[76] Zhang RG, Li GY, Wang XL. et al. TEsorter: an accurate and fast method to classify LTR-retrotransposons in plant genomes. Hortic Res. 2022;9:uhac017 35184178 10.1093/hr/uhac017 PMC9002660

[77] Lowe TM, Eddy SR. tRNAscan-SE: a program for improved detection of transfer RNA genes in genomic sequence. Nucleic Acids Res. 1997;25:955–64 9023104 10.1093/nar/25.5.955 PMC146525

[78] Kalvari I, Argasinska J, Quinones-Olvera N. et al. Rfam 13.0: shifting to a genome-centric resource for non-coding RNA families. Nucleic Acids Res. 2018;46:D335–42 29112718 10.1093/nar/gkx1038 PMC5753348

[79] Jones P, Binns D, Chang HY. et al. InterProScan 5: genome-scale protein function classification. Bioinformatics. 2014;30:1236–40 24451626 10.1093/bioinformatics/btu031 PMC3998142

[80] Simão FA, Waterhouse RM, Ioannidis P. et al. BUSCO: assessing genome assembly and annotation completeness with single-copy orthologs. Bioinformatics. 2015;31:3210–2 26059717 10.1093/bioinformatics/btv351

[81] Rhie A, Walenz BP, Koren S. et al. Merqury: reference-free quality, completeness, and phasing assessment for genome assemblies. Genome Biol. 2020;21:245 32928274 10.1186/s13059-020-02134-9 PMC7488777

[82] Guan J, Zhang J, Gong D. et al. Genomic analyses of rice bean landraces reveal adaptation and yield related loci to accelerate breeding. Nat Commun. 2022;13:5707 36175442 10.1038/s41467-022-33515-2 PMC9523027

[83] Lonardi S, Munoz-Amatriain M, Liang Q. et al. The genome of cowpea (*Vigna unguiculata* [L.] Walp.). Plant J. 2019;98:767–82 31017340 10.1111/tpj.14349 PMC6852540

[84] Quinlan AR . BEDTools: the Swiss-army tool for genome feature analysis. Curr Protoc Bioinformatics. 2014;47:11.12.1–34 10.1002/0471250953.bi1112s47 PMC4213956 25199790

[85] Chen S, Zhou Y, Chen Y. et al. Fastp: an ultra-fast all-in-one FASTQ preprocessor. Bioinformatics. 2018;34:i884–90 30423086 10.1093/bioinformatics/bty560 PMC6129281

[86] Patro R, Duggal G, Love MI. et al. Salmon provides fast and bias-aware quantification of transcript expression. Nat Methods. 2017;14:417–9 28263959 10.1038/nmeth.4197 PMC5600148

[87] Soneson C, Love MI, Robinson MD. Differential analyses for RNA-seq: transcript-level estimates improve gene-level inferences. F1000Res. 2015;4:1521 26925227 10.12688/f1000research.7563.1 PMC4712774

[88] Love MI, Huber W, Anders S. Moderated estimation of fold change and dispersion for RNA-seq data with DESeq2. Genome Biol. 2014;15:550 25516281 10.1186/s13059-014-0550-8 PMC4302049

[89] Li H . Aligning sequence reads, clone sequences and assembly contigs with BWA-MEM. 2013; *arXiv preprint arXiv* 1303: 3997

[90] Garrison E, Marth G. Haplotype-based variant detection from short-read sequencing. 2012; *arXiv preprint arXiv* 1207: 3907

[91] Cleal K, Baird DM. Dysgu: efficient structural variant calling using short or long reads. Nucleic Acids Res. 2022;50:e53 35100420 10.1093/nar/gkac039 PMC9122538

[92] Li H . Tabix: fast retrieval of sequence features from generic TAB-delimited files. Bioinformatics. 2011;27:718–9 21208982 10.1093/bioinformatics/btq671 PMC3042176

[93] Yang J, Lee SH, Goddard ME. et al. GCTA: a tool for genome-wide complex trait analysis. Am J Hum Genet. 2011;88:76–82 21167468 10.1016/j.ajhg.2010.11.011 PMC3014363

[94] Kopelman NM, Mayzel J, Jakobsson M. et al. Clumpak: a program for identifying clustering modes and packaging population structure inferences across *K*. Mol Ecol Resour. 2015;15:1179–91 25684545 10.1111/1755-0998.12387 PMC4534335

[95] Villanueva RAM, Chen ZJ. ggplot2: Elegant Graphics for Data Analysis. Taylor & Francis; Springer. 2019:160–7

[96] Danecek P, Auton A, Abecasis G. et al. The variant call format and VCFtools. Bioinformatics. 2011;27:2156–8 21653522 10.1093/bioinformatics/btr330 PMC3137218

[97] Chen H, Patterson N, Reich D. Population differentiation as a test for selective sweeps. Genome Res. 2010;20:393–402 20086244 10.1101/gr.100545.109 PMC2840981

[98] Wu T, Hu E, Xu S. et al. clusterProfiler 4.0: a universal enrichment tool for interpreting omics data. Innovation (Camb). 2021;2:100141 34557778 10.1016/j.xinn.2021.100141 PMC8454663

[99] Yu G . Enrichplot: visualization of functional enrichment result. 2021; *R Package Version* 1: 1

